# Paper mills as a reflection of the decline of scientific practices in our country

**DOI:** 10.17843/rpmesp.2023.404.13447

**Published:** 2023-12-18

**Authors:** Lely Solari, César Cabezas

**Affiliations:** 1 Instituto Nacional de Salud, Lima, Peru. Instituto Nacional de Salud Lima Peru

An important debate has arisen during the last few weeks as a result of a research paper mill being exposed by mass media outlets. The good and bad aspects of scientific publication were discussed initially, then the debate aimed at interventions for strengthening research capacities worldwide. First, we must acknowledge that paper mills represent a setback for good research practice and a lack of compliance with ethical standards in research, since scientific publication is the last and fundamental step in the research process (without dissemination, science cannot reach its potential impact). However, this phenomenon should also be seen as part of a broader process of institutional deterioration in several social sectors, particularly the education sector, which extends from the poorest inhabitants to the highest representation levels in the country. Buying authorship in scientific publications to climb up the research rank system could be considered to be a denaturalization of the process of obtaining degrees without meeting the minimum requirements. Selling authorship, on the other hand, could be considered an illegal ramification of certifying professionals who do not meet the requirements, or who fail the minimum professional sufficiency exams, just to receive payment.

On the specific issue of scientific publication practices, we remind the research community that there are international regulations that determine the minimum standards that regular publication processes should have. At the level of medical sciences, the International Committee of Medical Journal Editors (ICMJE) ^1^ recommends the general processes that scientific journals should follow to ensure correct scientific publications. The editorial policy of the Revista Peruana de Medicina Experimental y Salud Pública (RPMESP) adheres to these recommendations and translates them into standardized procedures ^2^, which, although they may be rigid for some authors, are important to avoid transgressions to the editorial process. On the other hand, the guidelines of the Committee on Publication Ethics (COPE) ^3^ establish specific guidelines to be taken into account when an ethical transgression occurs during the editorial process, which we have had to resort to on some occasions and which have been an invaluable guide. In addition, we should consider that there are documents that systematize initiatives aimed at strengthening scientific publication ^4^.

We should consider that, even if these processes are rigorously followed, various types of ethical misconduct can occur during the publishing process, many of which may go unnoticed. The paper mill cases we have seen are only the most notorious and easily detectable, but also those that should be corrected in the first place. Most irregular situations can be prevented by reinforcing safeguards, but proven misconducts need to be investigated and sanctions must be applied.

In this context, we call on the local scientific community to maintain the common goal of developing and strengthening science in our country. In Peru, scientific activity is led by the National Council for Science, Technology and Technological Innovation (CONCYTEC), the institution responsible for reviewing the best interventions to solve the problems we are facing and which, accordingly, should be implemented. The paper mill crisis should not be a pretext to remember and enumerate all the problems we have been accumulating during decades of neglect of science by the government, but the opportunity to focus on implementing preventive and corrective measures to solve the problem that afflicts us. We, at RPMESP, commit ourselves to support the initiatives that CONCYTEC, having thoroughly verified their potential effectiveness, considers appropriate to implement. Likewise, we will adhere to other evidence-based measures aimed at strengthening our scientific publication system.

Finally, we have to adapt to the new times, in which dissemination of scientific information has changed and moves significantly to social networks, in which not only technological advances are disseminated, but also serve as starting points for scientific debate. Scientific publication - in all its platforms - is based on good faith and scientific integrity, which should be the essential spirit of research. We must not let the credibility of science be put at risk in an already weakened system such as ours. herefore, leaders in the fields of science in our country must not only instruct and provide us the example of being honest and responsible researchers, as many have been doing, but also show us with concrete actions that our common goal, which is to strengthen our research system, is more important than the opinions that differentiate us.
