# Supplementary material for: Paper mills as a reflection of the decline of scientific practices in our country
Source: Rev Peru Med Exp Salud Publica. 2023 Dec 18;40(4):390–1. doi: 10.17843/rpmesp.2023.404.13447 (PMC11138822; doi:10.17843/rpmesp.2023.404.13447)
Supplement: Translation

# Translation: La fábrica de artículos científicos como reflejo del deterioro de las prácticas científicas en nuestro país

**Authors:** Lely Solari, César Cabezas

**Affiliations:** 1 Instituto Nacional de Salud, Lima, Perú.

Durante las últimas semanas, a raíz de la exposición de una fábrica (granja) de artículos científicos en medios de comunicación masiva, se ha originado un importante debate, que inició con lo bueno y lo malo en la publicación científica pero que se ha ido generalizando a las intervenciones destinadas a fortalecer globalmente las capacidades de investigación. En primer lugar, se debe reconocer que la fabricación de artículos refleja un retroceso en las buenas prácticas y el respeto a los estándares éticos del proceso de investigación en nuestro medio, ya que la publicación científica es el último y fundamental paso de este proceso (sin difusión, la ciencia no alcanza su impacto potencial). Sin embargo, este fenómeno también se debe entender como parte de un proceso más extenso de deterioro institucional que viene ocurriendo en diversos ámbitos del quehacer social, sobre todo en el educativo, y que transcurre desde los pobladores más humildes hasta los niveles más altos de representación en el país. Comprar publicaciones científicas para escalar en el sistema de investigación podría verse como una variación desnaturalizada de la figura de obtener títulos sin haber cumplido con los requisitos indispensables. Venderlas, por otro lado, podría considerarse una ramificación ilegal del hecho de certificar a profesionales que no cumplen con los requisitos, o que desaprueban los exámenes mínimos de suficiencia profesional, solo para recibir el pago por titulación. En este contexto, esta llamada de atención a la comunidad científica constituye una oportunidad para mejorar, ya que, por el momento, estos casos son relativamente infrecuentes, y deberíamos trabajar para que disminuyan y desaparezcan.

En el tema específico de las prácticas en publicación científica, recordamos a la comunidad de investigadores que existen regulaciones internacionales que dictan los estándares mínimos que deben tener los procesos regulares de publicación. A nivel de ciencias médicas, existe el Comité Internacional de Editores de Revistas Médicas (ICMJE) ^1^, que recomienda los procesos generales que las revistas científicas deben de seguir para asegurar publicaciones científicas correctas. La política editorial de la Revista Peruana de Medicina Experimental y Salud Pública (RPMESP) se adhiere a estas recomendaciones y las traduce en procedimientos estandarizados ^2^, que, si bien pueden resultar rígidos para algunos autores, son importantes para evitar transgresiones al proceso editorial. Por otro lado, existen las guías del Comité de Ética en Publicación (COPE) ^3^ que establecen lineamientos específicos a tomar en cuenta cuando ocurre una transgresión ética durante el proceso editorial, y a las cuales hemos tenido que recurrir en algunas oportunidades y han constituido una guía invalorable. Además, se debe considerar que existen documentos que sistematizan las iniciativas destinadas a fortalecer la publicación científica ^4^.

Cabe manifestar que, incluso siguiendo rigurosamente estos procesos, pueden ocurrir diversos tipos de faltas éticas en la publicación, muchas de las cuales pueden pasar desapercibidas. Los casos que hemos visto en la granja de artículos son solo los más notorios y fácilmente detectables, pero también los que deben ser corregidos en primer lugar. Estamos seguros que, reforzando las salvaguardas se pueden prevenir la mayoría de situaciones irregulares, pero en los casos en los que se comprueben las faltas, es necesario que en el sistema de investigación existan sanciones proporcionales a estas malas prácticas o incluso delitos.

En este contexto, hacemos un llamado a la comunidad científica local a mantener el objetivo común del desarrollo y fortalecimiento de la ciencia en nuestro país. En el Perú, la actividad científica está liderada por el Consejo Nacional de Ciencia, Tecnología e Innovación Tecnológica (CONCYTEC), institución responsable de revisar cuáles son las mejores intervenciones para solucionar la problemática que estamos enfrentando y que, en concordancia, debe implementar. La crisis por la fabricación de artículos no debería ser un pretexto para hoy recordar y enumerar todos los problemas que hemos venido acumulando durante décadas de descuido de la ciencia por parte de los distintos gobiernos que hemos tenido, sino la oportunidad para concentrarnos en implementar las medidas preventivas y correctivas que solucionen el problema que nos aqueja. Desde la RPMESP, nos comprometemos a apoyar las iniciativas que CONCYTEC, habiendo verificado exhaustivamente su potencial efectividad, considere adecuado implementar. Asimismo, nos sumaremos a otras medidas, basadas en evidencia, que estén orientadas a fortalecer nuestro sistema de publicaciones científicas.

Por último, tenemos que adaptarnos a los nuevos tiempos, en los que las rutas de difusión de la información científica han cambiado y se están trasladando de manera significativa a las redes sociales, en las que no solo se divulgan los adelantos tecnológicos, sino en las que muchas veces se abre el debate científico. La publicación científica- en todas sus plataformas- se basa en la buena fe y la integridad científica, que debe ser el espíritu esencial de la investigación.

No debemos dejar que la credibilidad en la ciencia se ponga en riesgo, en un sistema ya debilitado como el nuestro. Por todo ello, es necesario que en nuestro país los líderes en ciencia, no sólo nos instruyan y nos den el ejemplo de ser investigadores probos y responsables, como muchos lo vienen haciendo, sino que también nos demuestren con acciones concretas que nuestro objetivo en común, que es fortalecer nuestro sistema de investigación, es más importante que las opiniones que nos diferencian.
